# Novel Immunoglobulin Domain Proteins Provide Insights into Evolution and Pathogenesis of SARS-CoV-2-Related Viruses

**DOI:** 10.1128/mBio.00760-20

**Published:** 2020-05-29

**Authors:** Yongjun Tan, Theresa Schneider, Matthew Leong, L. Aravind, Dapeng Zhang

**Affiliations:** a Department of Biology, College of Arts and Sciences, Saint Louis University, St. Louis, Missouri, USA; b School of Medicine, Saint Louis University, St. Louis, Missouri, USA; c National Center for Biotechnology Information, National Library of Medicine, National Institutes of Health, Bethesda, Maryland, USA; d Program of Bioinformatics and Computational Biology, College of Arts and Sciences, Saint Louis University, St. Louis, Missouri, USA; The Ohio State University

**Keywords:** coronavirus, COVID-19, SARS, ORF8, immunoglobulin, evolution, pathogenesis, immune evasion

## Abstract

A novel coronavirus, severe acute respiratory syndrome coronavirus 2 (SARS-CoV-2), was recently identified as the causative agent for the coronavirus disease 2019 (COVID-19) outbreak that has generated a global health crisis. We use a combination of genomic analysis and sensitive profile-based sequence and structure analysis to understand the potential pathogenesis determinants of this virus. As a result, we identify several fast-evolving genomic regions that might be at the interface of virus-host interactions, corresponding to the receptor binding domain of the Spike protein, the three tandem Macro fold domains in ORF1a, and the uncharacterized protein ORF8. Further, we show that ORF8 and several other proteins from alpha- and beta-CoVs belong to novel families of immunoglobulin (Ig) proteins. Among them, ORF8 is distinguished by being rapidly evolving, possessing a unique insert, and having a hypervariable position among SARS-CoV-2 genomes in its predicted ligand-binding groove. We also uncover numerous Ig domain proteins from several unrelated metazoan viruses, which are distinct in sequence and structure but share comparable architectures to those of the CoV Ig domain proteins. Hence, we propose that SARS-CoV-2 ORF8 and other previously unidentified CoV Ig domain proteins fall under the umbrella of a widespread strategy of deployment of Ig domain proteins in animal viruses as pathogenicity factors that modulate host immunity. The rapid evolution of the ORF8 Ig domain proteins points to a potential evolutionary arms race between viruses and hosts, likely arising from immune pressure, and suggests a role in transmission between distinct host species.

## Abstract (precis)

The ongoing COVID-19 pandemic strongly emphasizes the need for a more complete understanding of the biology and pathogenesis of its causative agent SARS-CoV-2. Despite intense scrutiny, several proteins encoded by the genomes of SARS-CoV-2 and other SARS-like coronaviruses remain enigmatic. Moreover, the high infectivity and severity of SARS-CoV-2 in certain individuals make wet-lab studies currently challenging. In this study, we used a series of computational strategies to identify several fast-evolving regions of SARS-CoV-2 proteins which are potentially under host immune pressure. Most notably, the hitherto-uncharacterized protein encoded by ORF8 is one of them. Using sensitive sequence and structural analysis methods, we show that ORF8 and several other proteins from alpha- and beta-coronavirus comprise novel families of immunoglobulin domain proteins, which might function as potential immune modulators to delay or attenuate the host immune response against the viruses.

## INTRODUCTION

Nidoviruses are a group of lipid-enveloped viruses with nonsegmented RNA genomes and are known to infect animals, including molluscs, arthropods, and vertebrates (1), and apparently also the oomycete *Plasmopara* (NCBI taxonomy ID 2692091). Among them are the coronaviruses (CoVs), which possess the largest known monopartite RNA genome and are classified into four genera—*Alphacoronavirus*, *Betacoronavirus*, *Gammacoronavirus*, and *Deltacoronavirus* (2). Over the past 2 decades, beta-CoVs, including the viruses responsible for severe acute respiratory syndrome (SARS) in 2003 and Middle Eastern respiratory syndrome (MERS) in 2012, and alpha-CoV, the swine acute diarrhea syndrome coronavirus (SADS-CoV) (3), have emerged as significant human and veterinary health concerns with major economic consequences (4, 5). Recently, a novel coronavirus (SARS-CoV-2) was identified as the causative agent of coronavirus disease 2019 (COVID-19), a severe respiratory disease that has been infecting humans since late 2019 (6, 7). Phylogenomic analysis has shown that it belongs to the same large clade of beta-CoVs as the original SARS-CoV, with a likely origin in bats (6, 8, 9). COVID-19 presents itself with an incubation period ranging from 1 to 24 days followed by the potential development of a constellation of symptoms, including fever, dry cough, fatigue, diarrhea, myalgia, lymphopenia, dyspnea, and bilateral ground-glass opacities in the lungs (10–12). In some patients, this can proceed to fatal respiratory failure, characterized by acute lung injury (13) and acute respiratory distress syndrome (12). In most moribund patients, COVID-19 is accompanied by an inflammatory cytokine storm (12), which suggests that virus-induced immunopathological events may play a role in the development of disease severity. Finally, COVID-19’s ability to be transmitted during asymptomatic stages (14) makes prevention challenging.

Due to the rapid global spread of COVID-19 and its extreme severity in certain individuals, the need for a more complete understanding of the biology and pathogenesis of SARS-CoV-2 has become critical. Despite intense scrutiny of COVID-19 and SARS-related viruses, multiple proteins encoded by their genomes remain enigmatic. These include ORF3a, ORF6, ORF8, ORF10, the M protein, and certain regions of the ORF1a polyprotein that contains multiple domains. Furthermore, the high infectivity of SARS-CoV-2 makes wet-lab studies especially challenging. For these reasons, computational analysis is an effective way to study potential functions of the proteins and pathogenesis mechanisms of SARS-CoV-2-related viruses, which can then guide directed *in vivo* experimental studies. In this study, we have identified several fast-evolving genomic regions corresponding to the N-terminal region of the ORF1a polyprotein, the Spike protein, and the uncharacterized protein encoded by ORF8. By using dedicated domain-centric sequence and structural analysis, we show that ORF8 and several proteins from alpha- and beta-coronavirus define novel families of immunoglobulin (Ig) domains, which might function as potential immune modulators to delay or attenuate the host immune response against the viruses.

## RESULTS AND DISCUSSION

### The evolutionary arms race and fast-evolving genomic regions.

In our previous work, we successfully discovered several distinct classes of biological conflict systems, including polymorphic toxin systems involved in bacterial interactions (15–17), CR effector systems at the interface of eukaryotic pathogen/symbiont-host interactions (18), nucleotide-centric conflict systems (19), and DNA modification systems deployed in bacteriophage-bacterium conflicts (20). A common principle behind these disparate systems is that the parties locked in the biological conflict constantly evolve new offensive or defensive mechanisms in order to maintain an edge over the antagonist in the high-stakes battle, a concept termed the “evolutionary arms race.” For example, animals utilize a variety of rapidly evolving immunity molecules, often featuring immunoglobulin domains, for effective recognition and clearance of their viral pathogens. The viruses in turn respond by evolving a series of countermeasures to evade or hijack the host immune system. Therefore, the race between host and virus is never-ending, involving cycles of adaptation, in which the “winners” and “losers” frequently swap positions. On this basis, we reason that genomic regions characterized as being fast-evolving, subject to recombination between different virus strains, or newly introduced from other genomes would potentially contribute to the pathogenicity of the virus. Thus, uncovering signals of evolutionary arms races in genome and protein sequences can potentially help identify molecules involved in the pathogenesis of viruses like SARS-CoV-2.

### Comparative genomics unveils fast-evolving genomic regions of SARS-CoV-2-related viruses.

**FIG 1:**
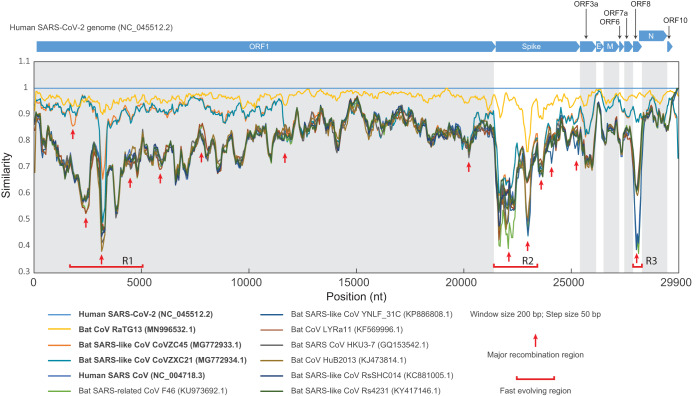
Genome similarity analysis of SARS-CoV-2-related viruses. The similarity plot of SARS-CoV-2-related CoVs compared to the human SARS-CoV-2 Wuhan-Hu-1 genome (GenBank accession no. NC_045512.2) is drawn based on a multiple-sequence alignment of the whole genomes. Each point represents percent identity of a 200-bp window of the alignment with a 50-bp step size between the points in each pair. The open reading frames of the SARS-CoV-2 genome (NC_045512.2) are shown above the plot. Each colored line corresponds to the nucleotide similarity between the human SARS-CoV-2 genome and the respective other CoV genome. The recombination events are represented by dispersed regions (indicated by red arrows), which deviate from the overall pattern of similarity between genomes, while the fast-evolving regions are represented by valleys where there is low similarity between genome regions (R1, R2, and R3). An in-depth analysis performed with various sizes of similarity windows is shown in Fig. S1. For detailed information about the genomes that were used in this study, refer to Table S1.

To identify potential anomalously diverging genomic regions in SARS-CoV-2, we conducted a sequence similarity scan of SARS-related coronavirus genomes. Similarity plots show that the bat CoV RaTG13 is the closest relative of SARS-CoV-2, with no evidence for recombination between them (Fig. 1; see also Fig. S1 in the supplemental material). SARS-CoV-2 also shows high similarity to two other bat viruses, CoVZXC21 and CoVZXC45—first in the 5′ half of ORF1 and again after nucleotide position 20000 of the genome. However, the remaining parts of ORF1 of SARS-CoV-2 and RatG13 show no specific relationship to these two bat viruses. This suggests that a recombination event due to template switching by the RNA polymerase occurred between the common ancestor of SARS-CoV-2 and RaTG13 and probably another member of the SARS-related clade close to CoVZXC21 and CoVZXC45. In addition to this major recombination event, by examining similarity windows of various sizes, we identified and refined several minimum regions which might have undergone recombinational diversification during the emergence of SARS-CoV-2 (Fig. S1). They are presented by dispersed regions (indicated by red arrows), deviating from the overall pattern of similarity between genomes. Furthermore, the similarity plot reveals several regions (R1, R2, and R3) displaying deep valleys with low sequence similarity between related genomes, indicating that such regions are evolving quickly through diversifying mutations. Each region also contains several potential recombination signals, indicating that recombination might also contribute to the diversification (Fig. 1; see also Fig. S1).

10.1128/mBio.00760-20.1 FIG S1 Genome comparison analysis of SARS-CoV-2-related viruses. The similarity plot of SARS-CoV-2-related CoVs compared to the human SARS-CoV-2 Wuhan-Hu-1 genome (GenBank accession no. NC_045512.2) is drawn based on a multiple-sequence alignment of the whole genomes. Each point represents a different slicing window size from the alignment with a different step size between the points in each pair. For each plot, the window size and step size are shown at the top left. Horizontal bars above the top plot correspond to the different open reading frames of the SARS-CoV-2 genome (NC_045512.2). Each differently colored line corresponds to the nucleotide similarity between the human SARS-CoV-2 genome (NC_045512.2) and the respective CoV genome. The red arrows and solid lines surround regions which display major recombination within the SARS-CoV-2-related CoV genomes. The single red arrows point to specific regions of recombination. Download FIG S1, PDF file, 1.1 MB. Copyright © 2020 Tan et al. 2020 Tan et al. This content is distributed under the terms of the Creative Commons Attribution 4.0 International license.

10.1128/mBio.00760-20.5 TABLE S1 Detailed information of human SARS-CoV-2 Wuhan-Hu-1 genome and other related CoV genomes that were used in this study (Fig. 1; see also Fig. S1). Download Table S1, DOCX file, 0.02 MB. Copyright © 2020 Tan et al. 2020 Tan et al. This content is distributed under the terms of the Creative Commons Attribution 4.0 International license.

Notably, the R2 region encodes the extracellular part of the Spike protein and one of the deep valleys corresponds to the receptor binding domain (RBD) of this protein. The Spike RBD is the region interacting with the human receptor, ACE2 (21, 22); therefore, its rapid evolution might have facilitated the development of a high affinity to ACE2. This provides a proof of concept that our computational analysis is effective in identifying the potential genomic regions that are situated at the interface of host and viral interactions. Another fast-evolving region, R1 (nucleotides [nt] 3000 to 5000), overlaps the stretch of the genome coding for residues 1000 to 1500 of ORF1a, which contains 3 tandem copies of the Macro fold domain. Macro domains bind NAD^+^-derived ADP-ribose (ADPr) derivatives and catalyze their processing or hydrolysis (23). The three Macro domains might be involved in RNA end processing and in countering ADPr signals, which may be deployed by the host as part of the innate immune response against viruses (24). The third striking region, R3, corresponds to the protein encoded by ORF8, whose function in SARS-like coronaviruses has remained a mystery for several years (25).

### ORF8 and several other CoV proteins comprise novel immunoglobulin families.

**FIG 2:**
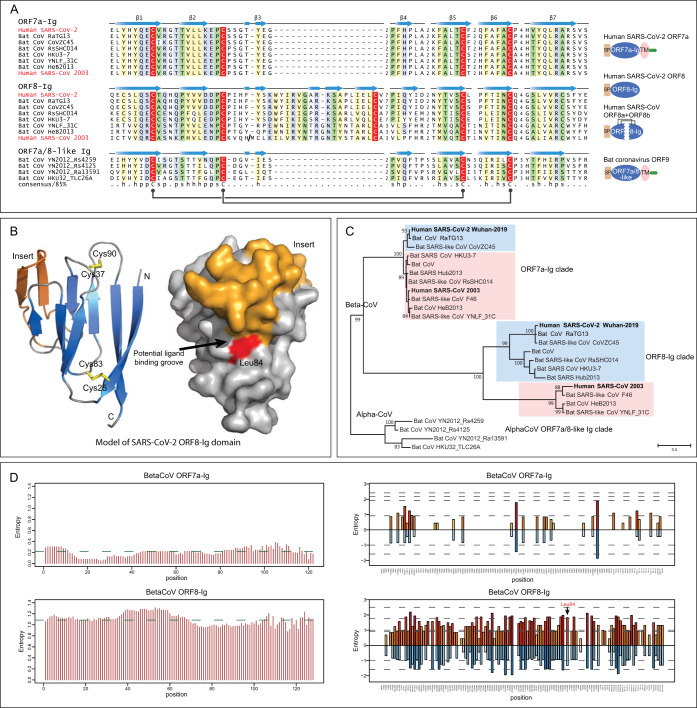
Sequence, structure, and evolutionary analysis of novel Ig domain proteins in SARS-CoV-2-related CoVs. (A) Multiple-sequence alignment (MSA) and representative domain architectures of ORF7a-Ig, ORF8-Ig, and ORF7a/8-like Ig domain families. Each sequence in the MSA is labeled by its species abbreviation followed by its source. The predicted secondary structure is shown above each alignment, and the consensus is shown below the superalignment, where “h” stands for hydrophobic residues, “s” for small residues, and “p” for polar residues. Two pairs of conserved cysteines that form disulfide bonds are highlighted in red. (B) Homology model of the SARS-CoV-2 ORF8-Ig domain (GenBank accession no. YP_009724396.1) and the location of the hypervariable position corresponding to Leu84 in the predicted ligand-binding groove. The β-sheets of the common core of the Ig fold are colored in blue, the insertion in ORF8-Ig in orange, and the loops in gray. The characteristic disulfide bonds are highlighted in yellow. (C) Maximum likelihood phylogenetic analysis of CoV Ig domain families. Supporting values from 100 bootstraps are shown for the major branches only. (D) Entropy plot for the ORF7a and ORF8 proteins in betacoronavirus. (Left) Shannon entropy data were computed for each column for a character space of 20 amino acids and are presented as mean entropy in a sliding window of 30 residues. The mean entropy across the entire length of the protein is indicated as a green horizontal line. (Right) Shannon entropy data computed based on regular amino acid alphabet (20 amino acids) are shown above the zero line in shades of orange. Shannon entropy data computed based on a reduced alphabet of 8 residues are shown below the zero line in shades of blue. Where a position shows high entropy in both alphabets, it is a sign of potential positive selection at those positions for amino acids of different chemical characters.

The ORF8 protein is one of the so-called accessory proteins, which do not participate in viral replication (26, 27), raising the possibility that it might have a direct role in viral pathogenesis via interactions with host molecules. It is predicted to be a secreted protein with a theoretical molecular weight of approximately 12.22 kDa without the signal peptide. It is present only in some beta-CoVs, including SARS-CoV-2, but not in the MERS-like clade. Profile-profile comparisons using a sequence profile built from the multiple-sequence alignment (MSA) of all available ORF8 proteins showed it to be unexpectedly homologous to the membrane-anchored ORF7a protein from the same subset of beta-CoVs and to several proteins (variously annotated as ORF9 or ORF10) from a subset of bat alpha-CoVs (Fig. 2A) (probability = 94% of profile-profile match) (28). ORF7a is a known member of the immunoglobulin (Ig) domain superfamily, and a structure similarity search performed via DALI revealed that it is related to extracellular metazoan Ig domains that are involved in adhesion, such as ICAM (29, 30). The beta-CoV ORF8 and ORF7a and the alpha-CoV Ig domains display a classic β-sandwich fold with seven β-strands and share the characteristic pattern with metazoan Ig domains of two cysteines which form stabilizing disulfide bonds (Fig. 2A; see also Fig. S2) (31). They are unified as a clade by the presence of an additional pair of conserved disulfide-bonding cysteines (Fig. 2A). Nonetheless, there are notable structural differences between the three groups of proteins. ORF8 is distinguished from ORF7a and alpha-CoV Ig proteins by the loss of its C-terminal transmembrane (TM) helix and the acquisition of a long insertion between strands 3 and 4 with a conserved cysteine which might facilitate dimerization through disulfide-bond formation (Fig. 2A). The homology model of ORF8, based on the structure of SARS-CoV ORF7a (pdb identifier [id]: 1XAK), suggests that this insertion augments a potential peptide-ligand binding groove that has been proposed for ORF7a (Fig. 2B; see also Fig. S3). Hence, the emergence of the insertion is directly linked with the acquisition of a modified interaction interface.

10.1128/mBio.00760-20.2 FIG S2 Full-length multiple-sequence alignment of ORF7a, ORF8-Ig, and ORF7a/8-like proteins. Each sequence in the MSA is labeled by its species abbreviation followed by its isolation date and NCBI accession number. The predicted secondary structure is shown above the alignment, and the consensus is shown below the alignment, where “h” stands for hydrophobic residues, “s” for small residues, and “p” for polar residues. The characteristic signal peptide, the TM region, and a stretch of basic residues are also labeled. Download FIG S2, PDF file, 0.6 MB. Copyright © 2020 Tan et al. 2020 Tan et al. This content is distributed under the terms of the Creative Commons Attribution 4.0 International license.

10.1128/mBio.00760-20.3 FIG S3 Structural analysis of SARS-CoV-2 Ig domains. Download FIG S3, PDF file, 2.6 MB. Copyright © 2020 Tan et al. 2020 Tan et al. This content is distributed under the terms of the Creative Commons Attribution 4.0 International license.

**FIG 3:**
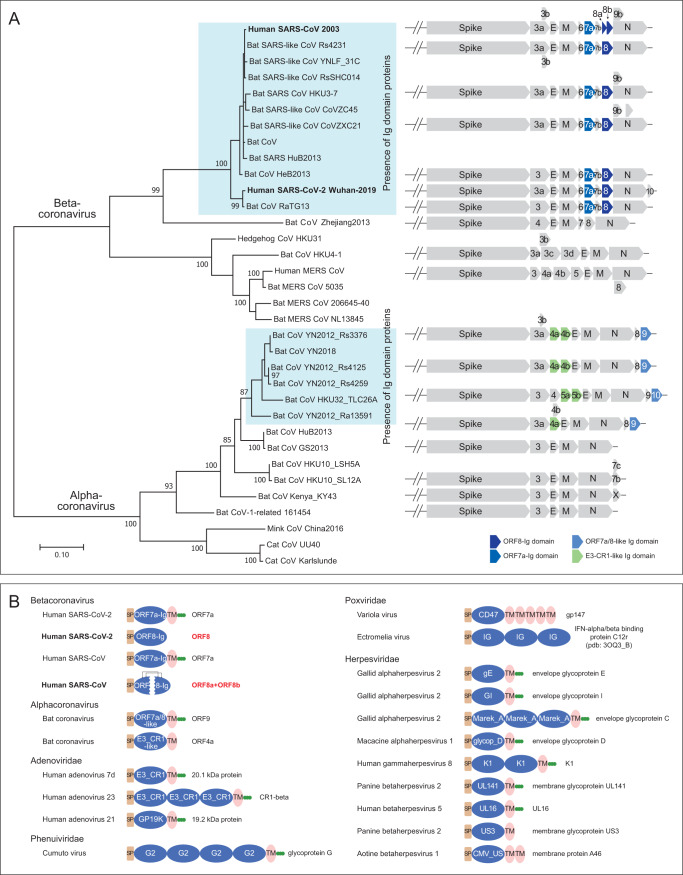
(A) Phylogenetic relationship and genomic organization of SARS-CoV-2-related CoVs. The tree of coronaviruses was built based on an MSA of a coronavirus RNA-directed, RNA polymerase domain using a maximum likelihood model. Supporting values from 100 bootstraps are shown for the major branches only. The genomic regions coding for the spike and other accessory proteins are illustrated at the right with respect to the terminal clade of the phylogenetic tree. The blue/green shade highlights the four Ig domain families that were identified in this study. (B) Representative domain architectures of the Ig components in different animal viruses. Proteins were grouped based on their families, except for proteins of coronavirus, which were grouped based on their genus. For the NCBI accession numbers, refer to the Table S2.

In addition to these families, we identified a fourth family of Ig proteins from the same alpha-CoVs which contained the ORF7a/8-like Ig family discussed above (Fig. 3A). These alpha-CoVs typically possess one or two paralogous copies annotated as either ORF4a/b or NS5a/b. According to their sequences, these Ig domains are not closely related to the ORF7a and ORF8 Ig domains (Fig. S4). However, profile-profile searches have shown that they are related to Ig domains found in the adenoviral E3-CR1 proteins (probability of 90% of matching the Pfam CR1 Ig domain profile) (Fig. S4). In these searches, they also yielded weaker hits to two other Ig domains, namely, the poxviral decoy interferon receptors (probability of 62.4%) and human T-cell surface CD3 zeta (probability of 51.5%) (Fig. S4).

10.1128/mBio.00760-20.4 FIG S4 Multiple-sequence alignment of alpha-CoV E3-CR1-like Ig domain proteins and other related Ig domains identified by profile-profile searches. Download FIG S4, PDF file, 1.2 MB. Copyright © 2020 Tan et al. 2020 Tan et al. This content is distributed under the terms of the Creative Commons Attribution 4.0 International license.

10.1128/mBio.00760-20.6 TABLE S2 Summary of representatives of viral Ig domain proteins and associated Pfam domains which were identified in this study. Download Table S2, DOCX file, 0.02 MB. Copyright © 2020 Tan et al. 2020 Tan et al. This content is distributed under the terms of the Creative Commons Attribution 4.0 International license.

### ORF8 is a fast-evolving protein in SARS-CoV-2-related viruses.

Phylogenetic analysis of the ORF7a and ORF8 and alpha-CoV Ig domains shows that each group represents a distinct clade (Fig. 2C). The tree topology of ORF7a mirrors that of the polymerase tree (Fig. 3A); however, the topology of the ORF8-Ig clade is not consistent with it. This might be due to a recombination event between the SARS-CoV-2-related CoVs (as suggested by the similarity plot analysis) and/or unusual divergence under selection. To better understand the functional difference between ORF8 and ORF7a, we examined the column-wise Shannon entropy (*H*) data in the 20-amino-acid alphabet and found that ORF8 has significantly higher mean entropy than ORF7a (ORF8, 1.09; ORF7a, 0.22 [*P* < 10^−16^ for the *H*_0_ of congruent means by *t* test]) (Fig. 2D). By comparing column-wise entropies in both the 20-amino-acid alphabet and a reduced 8-letter alphabet (where amino acids are grouped based on similar side chain chemistries), we found at least 14 positions in ORF8 which show high entropy in both alphabets compared to a single position in ORF7a (Fig. 2D). This indicates that ORF8 is a fast-evolving protein under selection for diversity, in contrast to ORF7a. Strikingly, one of these highly variable positions, which features residues with very different side chain characteristics (hydrophobic, acidic, alcoholic, and proline), corresponding to Leu84, was also identified as the most variable position across 54 closely related human SARS-CoV-2 genome sequences (32). In our structural model, this residue is positioned at the predicted peptide-ligand binding groove of the ORF8-Ig domain (Fig. 2B). Therefore, our entropy and structural analysis of the ORF8-Ig domain, in conjunction with its hypervariable position found in human SARS-CoV-2 genomes, points to a role of ORF8 at the interface of the host-virus interaction, possibly in a pathogenic context.

### Ig domain proteins are newly acquired in subsets of alpha- and beta-CoVs.

We examined the distribution of CoV Ig proteins in the context of a phylogenetic tree of both beta- and alpha-CoVs based on their polymerase proteins (Fig. 3A). Other than the two subsets of beta-CoVs and alpha-CoVs that contained the Ig domain proteins described above, no CoVs contained any Ig domain proteins (Fig. 3A). The immediate sister-groups of the Ig-containing CoVs typically had Spike, E, M, and N and one or two other uncharacterized accessory proteins which are not Ig domains to the best of our knowledge. Alpha-CoV ORF9 and ORF10 share a C-terminal TM helix and, along with ORF7a of the beta-CoVs, lack the insertion in the Ig domain (Fig. 2A). Hence, it is possible that this architecture represents the ancestral state which was present in the common ancestor of both alpha-CoVs and beta-CoVs. Under this scenario, the protein was displaced/lost in both certain alpha-CoVs and certain beta-CoVs. Alternatively, ORF7a could have been exchanged between alpha- and beta-CoVs. In both scenarios, ORF8 likely arose via a duplication of ORF7a in the beta-CoVs. Although we were unable to identify the ultimate precursors of the CoV Ig domains, they are likely to have been acquired on at least two independent occasions from different sources. The CoV ORF7a-ORF8 families might have been ultimately derived from the metazoan adhesion Ig families, and the ORF4a/b-like Ig domains of alpha-CoVs were potentially acquired from adenoviral CR1 Ig domains with which they share some specific sequence features. The latter case is reminiscent of the acquisition of a membrane fusion protein (p10) by the Rousettus bat coronavirus (a beta-CoV) from a vertebrate orthoreovirus with double-stranded RNA genomes (33).

### Divergent Ig proteins with comparable architectures are deployed by distinct viruses.

The presence of multiple Ig domains with different affinities in CoVs prompted us to more generally survey animal viruses for Ig domains. By using the Pfam hidden Markov models (HMMs) (34) and the HMMs and position-specific scoring matrices (PSSMs) (35) created from newly identified Ig domains, we were able to identify about 17 distinct viral Ig domain families in a wider diversity of animal viruses (Fig. 3B; see also Table S2 in the supplemental material). In addition to CoVs, such Ig domain proteins can be found in adenoviruses, nucleocytoplasmic large DNA viruses (NCLDVs), herpesviruses, and phenuiviruses. These viral Ig domains are highly divergent; many of them are found only in certain viral groups. However, the majority have an architecture comparable to the CoV-Ig domains, with an N-terminal signal peptide, one or multiple Ig domains, and a C-terminal TM region often followed by a stretch of basic residues. Thus, although the Ig domains are not a universally present component of animal viruses, they have been acquired and retained independently by a wide range of animal viruses. The presence of a proofreading 3′–5′ exonuclease has been proposed to favor the emergence of larger RNA genomes in CoVs (36). Indeed, this might have also contributed to the acquisition of potential pathogenesis factors such as the Ig domains described here which are comparable to those seen in DNA viruses such as adenoviruses and NCLDVs (37).

### Novel CoV Ig domain proteins are potential immune modulators.

Why have diverse viruses independently acquired the Ig domain during their evolution? First, the Ig domains are major mediators of adhesive interactions in both eukaryotes and prokaryotes (31, 38, 39). Thus, this domain can be used for adherence for cell-to-cell spread (e.g., herpesviral Ig domain proteins) (40). Second, Ig domains are major building blocks of metazoan immune systems. Thus, viruses often utilize this domain to disrupt immune signaling of the host. For example, in adenoviruses, the CR1 Ig domain proteins have been shown to inhibit the surface expression of class I major histocompatibility complex (MHC) molecules by blocking their trafficking from the endoplasmic reticulum (ER) to the Golgi compartments (41) in infected cells. This has been shown to affect the host inflammatory response and to modulate the presentation of viral antigens to T cells (42). In mammalian poxviruses, the secreted Ig domain proteins function as interferon receptors or decoys that bind interferon-α/β and disrupt signaling via endogenous host receptors (43). Further, SARS-ORF7a has been implicated in an interaction with bone marrow stromal antigen 2 (BST-2), which tethers budding virions to the host cell in a broad-spectrum antiviral response, to prevent the N-linked glycosylation of BST-2, thereby crippling the host response against the virus (44). Given their shared evolutionary history and similar sequence and structural features, we propose that the newly identified CoV Ig domain proteins, such as ORF8 of SARS-CoV-2, might similarly function as immune modulators.

While ORF8 is a paralog of ORF7a, its lack of the TM segment, unique insertion pattern, and significantly more rapid evolution than the latter suggest that it is under selection—perhaps for interacting with a similarly variable host molecule or due to direct immune recognition by the host. One possible mechanism is that, like the adenoviral CR1 proteins, ORF8 interferes with variable MHC molecules to attenuate antigen presentation, resulting in ineffective detection of the virus by the host immune system. Consistent with this prediction of being at the interface of a virus-host interaction, several polymorphisms have been reported in the ORF8 region of the genome both over the course of the SARS epidemic and during the current COVID-19 pandemic. While ORF8 of SARS-CoV isolates from civets and early stages of the human SARS epidemic is intact, it split into two ORFs (ORF8a and ORF8b) due to a 29-nt deletion during the middle phase of the human SARS epidemic (25, 45). Further, genomic deletions totaling up to 415 nt were observed in the SARS-CoV ORF8 region in the very late phase of the SARS epidemic (46). While the ORF8a and ORF8b fragments have been proposed to form a complex in a yeast-two hybrid interaction study (47), other studies indicate that this deletion considerably reduces the fitness of SARS-CoV (46). Remarkably, a recent study of patients in Singapore pointed to a 382-nt deletion in SARS-CoV-2 (48), suggesting parallel disruption of this gene during the COVID-19 pandemic. Thus, instances of polymorphism in separate SARS-related viruses suggest that this protein might be a key determinant of the severity of the disease and might play a role in the differential virulence of the virus in different host types.

In conclusion, we have identified several fast-evolving regions in the SARS-CoV-2 genome, corresponding to the three Macro domains in ORF1a, the RBD in Spike, and the ORF8 protein, which might be participants in the host-pathogen arms race. We demonstrate that ORF8 is a hitherto-unrecognized immunoglobulin protein which shares general structural features with other Ig domain proteins from animal viruses. We propose that the fast-evolving ORF8 is a potential pathogenicity factor that might attenuate the host immune response and might have mutated during transmission between different hosts. We hope that the discovery and analyses of the novel Ig domain proteins reported here will help the community better understand the evolution and pathogenesis mechanisms of these coronaviruses.

## MATERIALS AND METHODS

### Genome comparison analysis.

We retrieved the SARS-CoV-2-related CoV genomes by searching against the nonredundant (nr) nucleotide database of the National Center for Biotechnology Information (NCBI) with the SARS-CoV-2 genome sequence (GenBank accession no. NC_045512.2) as a query (35). The program CD-HIT was used for similarity-based clustering (49). A multiple-sequence alignment (MSA) of whole-virus genomes was performed by the use of KALIGN (50). Based on the MSA, a similarity plot was constructed by a custom Python script, which calculated the identity between each subject sequence and the SARS-CoV-2 genome sequence based on a custom sliding window size and step size. Open reading frames of virus genomes used in this study were extracted from an NCBI GenBank file.

### Protein sequence analysis.

To collect protein homologs, iterative sequence profile searches were conducted by the programs PSI-BLAST (position-specific iterated BLAST) (35) and JACKHMMER (51), which searched against the nonredundant (nr) protein database of NCBI, with a cutoff E value of 0.005 serving as the significance threshold. Similarity-based clustering was conducted by BLASTCLUST, a BLAST score-based single-linkage clustering method (ftp://ftp.ncbi.nih.gov/blast/documents/blastclust.html). Multiple-sequence alignments were built using the KALIGN (50), MUSCLE (52), and PROMALS3D (53) programs, followed by careful manual adjustments based on the profile-profile alignment, the secondary structure information, and the structural alignment. Profile-profile comparisons were conducted using the HHpred program (28). The consensus of the alignment was calculated using a custom Perl script. The alignments were colored using an in-house alignment visualization program written in Perl and further modified using Adobe Illustrator. Signal peptides were predicted using the SignalP-5.0 server (54). The transmembrane regions were predicted using TMHMM Server v. 2.0 (55). The theoretical molecular weight of ORF8 without the N-terminal signal peptide was predicted based on the Compute pI/*M*_w_ server on ExPASy (https://web.expasy.org/compute_pi/).

### Identification of distinct viral Ig domain proteins.

By using the HHsearch program (56), we searched with several candidate Ig domains against the Pfam domain database (34), which generated a collection of distinct Ig domains. The HMMs and PSSMs of both the identified Pfam domains and our Ig domains were used to identify the Ig homologs in viral genomes using the hmmscan program of the HMMER package (57) and RPS-BLAST (35) with an E value cutoff of 0.001. The Pfam domain information can be found in the Table S2 in the supplemental material. The alignments of the newly identified Ig domains which were used to generate HMMs and PSSMs can be found in Data Set S1 in the supplemental material.

10.1128/mBio.00760-20.7 DATA SET S1 Sequence alignments of the newly identified CoV Ig domains. Download Data Set S1, TXT file, 0.01 MB. Copyright © 2020 Tan et al. 2020 Tan et al. This content is distributed under the terms of the Creative Commons Attribution 4.0 International license.

### Molecular phylogenetic analysis.

The evolutionary history was inferred by using the maximum likelihood method based on the JTT w/freq. model (58). The tree with the highest log likelihood is shown. Supporting values from 100 bootstraps are shown next to the branches (59). The initial tree(s) for the heuristic search was obtained automatically by applying Neighbor-Join and BioNJ algorithms to a matrix of pairwise distances estimated using a JTT model and then selecting the topology with the superior log likelihood value. A discrete Gamma distribution was used to model evolutionary rate differences among sites (4 categories). The rate variation model allowed for some sites to be evolutionarily invariable. The tree is drawn to scale, with branch lengths measured in the number of substitutions per site. The tree diagram was generated using MEGA Tree Explorer (60)

### Entropy analysis.

Position-wise Shannon entropy (*H*) for a given multiple-sequence alignment was calculated using the following equation:

$$H=-\sum_{i=1}^{M}P_{i}\log_{2}P_{i}$$

where *P* is the fraction of residues of amino acid type *i* and *M* is the number of amino acid types. The Shannon entropy value for the *i*th position in the alignment ranges from 0 (only one residue at that position) to 4.32 (all 20 residues equally represented at that position). Analysis of the entropy values which were thus derived was performed using the R language.

### Protein structure prediction and analysis.

The secondary structural prediction was conducted using the Jnet (Joint Network) program (61). Jnet is a neural-network-based predictor which trains neural networks from three different types of profiles: profile PSSM, profile HMM, and residue frequency profile. It generates a consensus secondary structure with an average accuracy of 72% or greater. The Modeller9v1 program (62) was utilized for homology modeling of the structure of SARS-CoV-2 ORF8 using SARS-CoV ORF7a (1xak_A) (63) as a template. The sequence identity between the SARS-CoV-2 ORF8 Ig and the SARS-CoV ORF7a Ig is 13%. Since sequence alignment is the most important factor affecting the quality of the model in such low-sequence-identity cases (64), the alignments used in this study were carefully built and cross-validated based on the information from HHpred and edited manually using the secondary structure information. Five models were generated and further refined using the ReFOLD server (65); the one that had the highest model accuracy *P* value (0.09) and a global model quality score of 0.33 was selected for further analysis. It should be noted that the model quality in this range is typical for models with a level of sequence identity to the template below 15% and containing an insertion without a corresponding template. However, the model, taken together with structural inferences drawn from sequence analysis, serves as a reasonable guide to analyze the major features of the domain and accurately captures elements such as disulfide bond constraints. Structural analyses and comparisons were conducted using the molecular visualization program PyMOL (66). The structural similarity search was performed using the DALI server (67).
