# Supplementary material for: Novel Immunoglobulin Domain Proteins Provide Insights into Evolution and Pathogenesis of SARS-CoV-2-Related Viruses
Source: mBio. 2020 May 29;11(3):e00760-20. doi: 10.1128/mBio.00760-20 (PMC7267882; doi:10.1128/mBio.00760-20)
Supplement: FIG S1 [file mBio.00760-20-sf001.pdf]

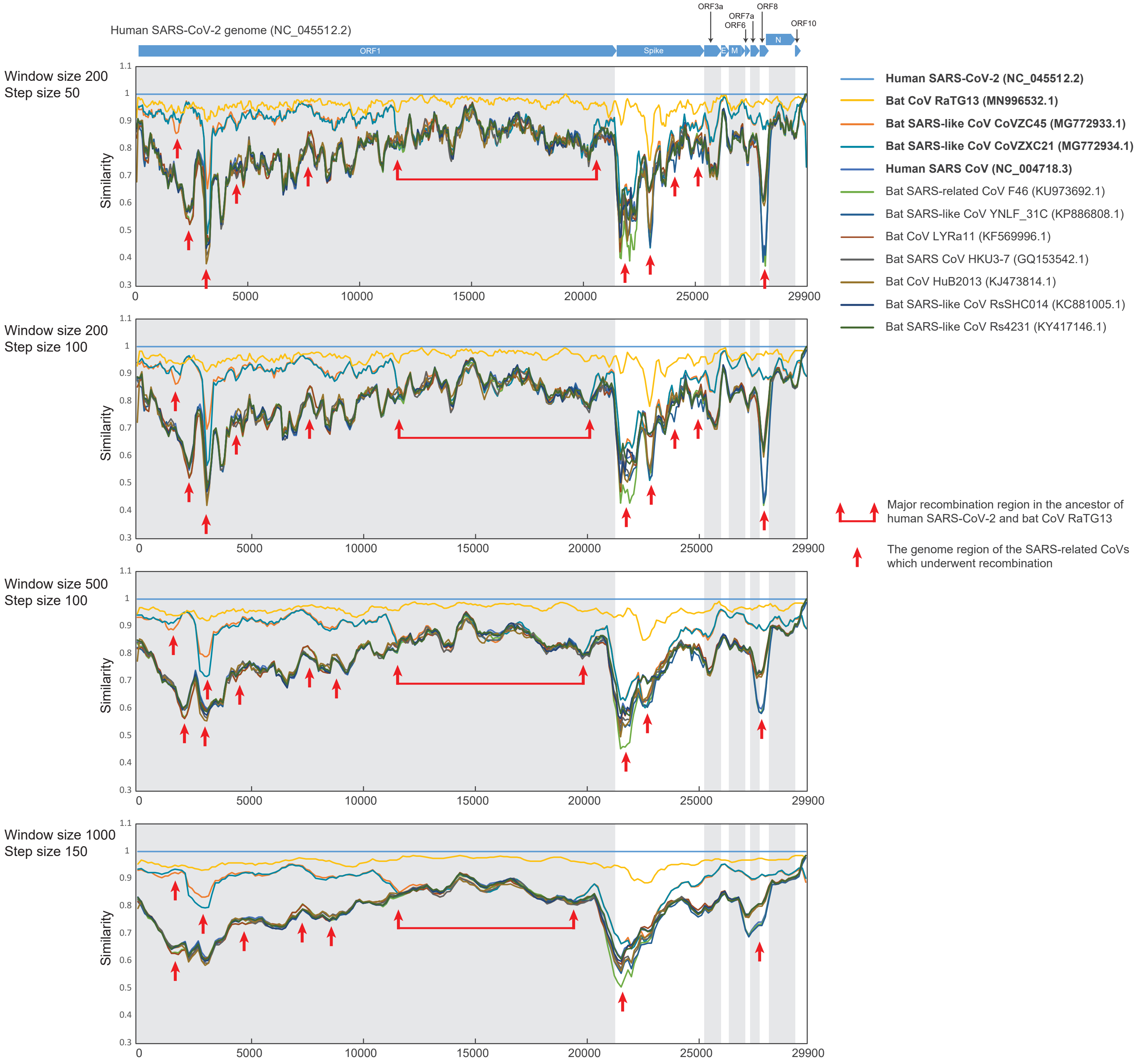
Human SARS-CoV-2 genome (NC_045512.2)
ORF1
Spike
ORF3a
ORF7a
ORF6
ORF8
ORF10
E
M
N
Window size 200
Step size 50
Window size 200
Step size 100
Window size 500
Step size 100
Window size 1000
Step size 150
Similarity
1.1
1
0.9
0.8
0.7
0.6
0.5
0.4
0.3
0
5000
10000
15000
20000
25000
29900
Human SARS-CoV-2 (NC_045512.2)
Bat CoV RaTG13 (MN996532.1)
Bat SARS-like CoV CoVZC45 (MG772933.1)
Bat SARS-like CoV CoVZXC21 (MG772934.1)
Human SARS CoV (NC_004718.3)
Bat SARS-related CoV F46 (KU973692.1)
Bat SARS-like CoV YNLF_31C (KP886808.1)
Bat CoV LYRa11 (KF569996.1)
Bat SARS CoV HKU3-7 (GQ153542.1)
Bat CoV HuB2013 (KJ473814.1)
Bat SARS-like CoV RsSHC014 (KC881005.1)
Bat SARS-like CoV Rs4231 (KY417146.1)
Major recombination region in the ancestor of human SARS-CoV-2 and bat CoV RaTG13
The genome region of the SARS-related CoVs which underwent recombination
