# Supplementary material for: Novel Immunoglobulin Domain Proteins Provide Insights into Evolution and Pathogenesis of SARS-CoV-2-Related Viruses
Source: mBio. 2020 May 29;11(3):e00760-20. doi: 10.1128/mBio.00760-20 (PMC7267882; doi:10.1128/mBio.00760-20)
Supplement: TABLE S1 [file mBio.00760-20-st001.docx]

**Table S1 Detailed information of Human SARS-CoV-2 Wuhan-Hu-1 genome and other SARS-related genomes that were used in this study (Fig. 1 & Fig. S1)**

| Organism | Host | NCBI ID | Year | Citation |
| --- | --- | --- | --- | --- |
| Severe acute respiratory syndrome coronavirus 2 (SARS-CoV-2) isolate Wuhan-Hu-1 | Human | NC_045512.2 | 2020 | A novel coronavirus associated with a respiratory disease in Wuhan of Hubei province, China (Unpublished) |
| Bat SARS-like coronavirus isolate bat-SL-CoVZC45 | Bat | MG772933.1 | 2018 | (1) |
| Bat SARS-like coronavirus isolate bat-SL-CoVZXC21 | Bat | MG772934.1 | 2018 | (1) |
| Bat coronavirus isolate RaTG13 | Bat | MN996532.1 | 2020 | Not Available |
| Severe acute respiratory syndrome-related coronavirus | Human | NC_004718.3 | 2003 | (2) |
| Severe acute respiratory syndrome-related coronavirus isolate F46 | Bat | KU973692.1 | 2017 | Identification of a new intermediate virus between bat-CoVs and SARS-CoVs from least horseshoe bats in China (Unpublished) |
| Bat SARS-like coronavirus YNLF_31C | Bat | KP886808.1 | 2015 | Not Available |
| Rhinolophus affinis coronavirus isolate LYRa11 | Bat | KF569996.1 | 2014 | (3) |
| Bat SARS coronavirus HKU3-7 | Bat | GQ153542.1 | 2010 | (4) |
| BtRs-BetaCoV/HuB2013 | Bat | KJ473814.1 | 2015 | (5) |
| Bat SARS-like coronavirus RsSHC014 | Bat | KC881005.1 | 2013 | (6) |
| Bat SARS-like coronavirus isolate Rs4231 | Bat | KY417146.1 | 2017 | (7) |

**REFERENCES**

1. Hu D, Zhu C, Ai L, He T, Wang Y, Ye F, Yang L, Ding C, Zhu X, Lv R. 2018. Genomic characterization and infectivity of a novel SARS-like coronavirus in Chinese bats. Emerging microbes & infections 7:1-10.

2. Marra MA, Jones SJ, Astell CR, Holt RA, Brooks-Wilson A, Butterfield YS, Khattra J, Asano JK, Barber SA, Chan SY. 2003. The genome sequence of the SARS-associated coronavirus. Science 300:1399-1404.

3. He B, Zhang Y, Xu L, Yang W, Yang F, Feng Y, Xia L, Zhou J, Zhen W, Feng Y. 2014. Identification of diverse alphacoronaviruses and genomic characterization of a novel severe acute respiratory syndrome-like coronavirus from bats in China. Journal of virology 88:7070-7082.

4. Lau SK, Li KS, Huang Y, Shek C-T, Tse H, Wang M, Choi GK, Xu H, Lam CS, Guo R. 2010. Ecoepidemiology and complete genome comparison of different strains of severe acute respiratory syndrome-related Rhinolophus bat coronavirus in China reveal bats as a reservoir for acute, self-limiting infection that allows recombination events. Journal of virology 84:2808-2819.

5. Wu Z, Yang L, Ren X, He G, Zhang J, Yang J, Qian Z, Dong J, Sun L, Zhu Y. 2016. Deciphering the bat virome catalog to better understand the ecological diversity of bat viruses and the bat origin of emerging infectious diseases. The ISME journal 10:609-620.

6. Ge X-Y, Li J-L, Yang X-L, Chmura AA, Zhu G, Epstein JH, Mazet JK, Hu B, Zhang W, Peng C. 2013. Isolation and characterization of a bat SARS-like coronavirus that uses the ACE2 receptor. Nature 503:535-538.

7. Hu B, Zeng L-P, Yang X-L, Ge X-Y, Zhang W, Li B, Xie J-Z, Shen X-R, Zhang Y-Z, Wang N. 2017. Discovery of a rich gene pool of bat SARS-related coronaviruses provides new insights into the origin of SARS coronavirus. PLoS pathogens 13.
