# Supplementary material for: Novel Immunoglobulin Domain Proteins Provide Insights into Evolution and Pathogenesis of SARS-CoV-2-Related Viruses
Source: mBio. 2020 May 29;11(3):e00760-20. doi: 10.1128/mBio.00760-20 (PMC7267882; doi:10.1128/mBio.00760-20)
Supplement: FIG S3 [file mBio.00760-20-sf003.pdf]

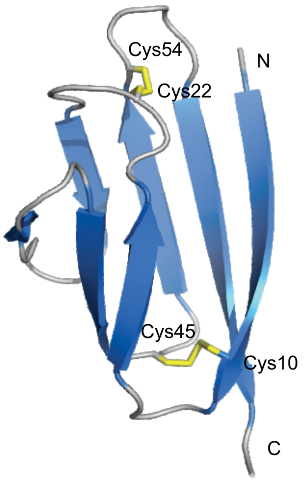

ORF7a-Ig domain
(PDB: 1xak_A)

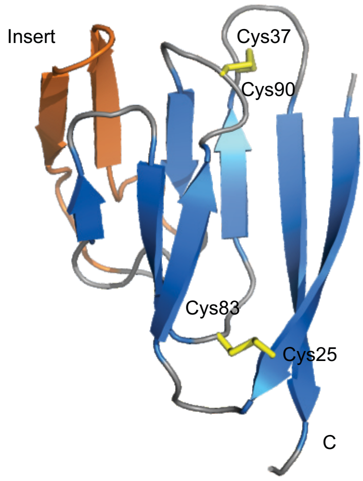

Model of ORF8-Ig domain

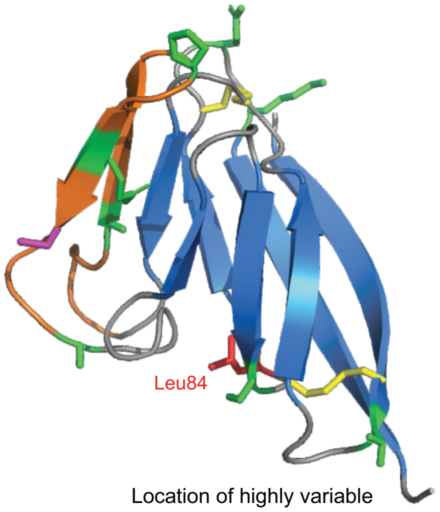

Location of highly variable
residues of ORF8-Ig domain
(sticks in green and red)

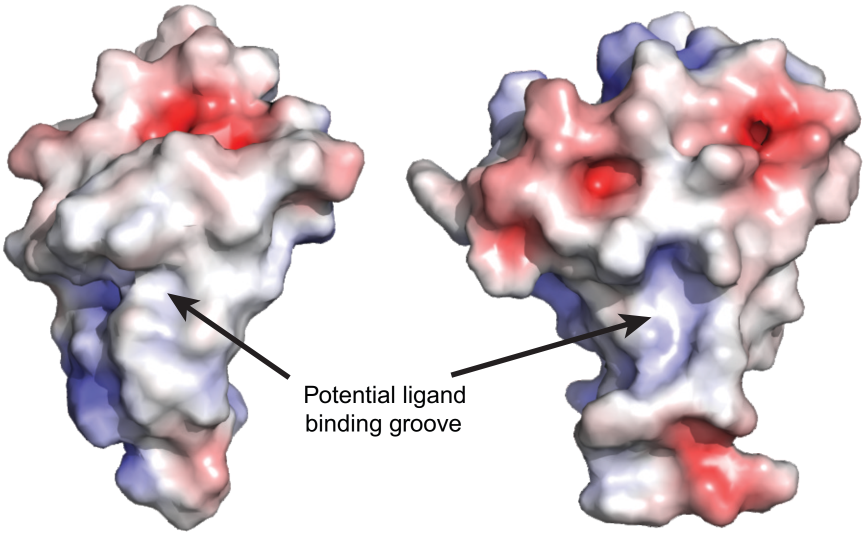

Surface view of ORF7a-Ig domain
(PDB: 1xak_A)

Surface view of ORF8-Ig domain

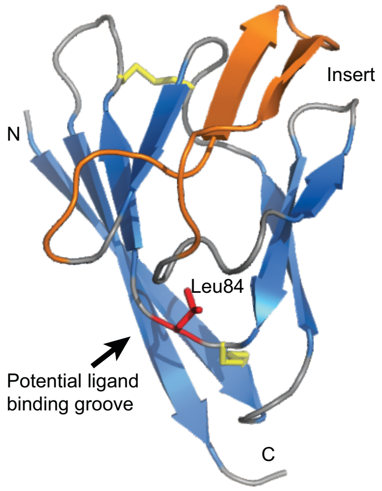

Location of hypervariable residue Leu84
on substrate-binding groove of the ORF8-ig domain
(stick in red)
