# Supplementary material for: Novel Immunoglobulin Domain Proteins Provide Insights into Evolution and Pathogenesis of SARS-CoV-2-Related Viruses
Source: mBio. 2020 May 29;11(3):e00760-20. doi: 10.1128/mBio.00760-20 (PMC7267882; doi:10.1128/mBio.00760-20)
Supplement: FIG S4 [file mBio.00760-20-sf004.pdf]

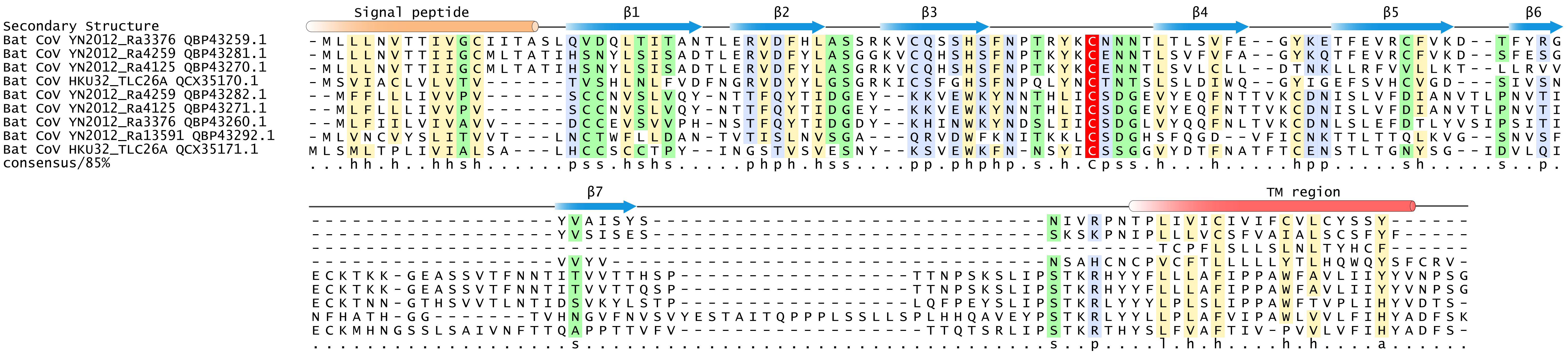

>PF02440.15 ; Adeno_E3_CR1 ; Adenovirus E3 region protein CR1
Probab=90.89 E-value=2.8 Score=29.56 Aligned_cols=38 Identities=18% Similarity=0.352 Sum_probs=0.0 Template_Neff=6.800

```
Q QBP43282.1        24  QYTIDGE-YKKVEWK----YNNTHLICSDGEVYEQFNTTVKCDNISLVF   67 (114)
Q Consensus         24  s~svd~~-y~rVdw~----~n~s~kICs~ghsfn~f~~~~kC~N~TLt~   67 (114)
                        .++..+. +..|.|+    -..+.++|.....+      |+|++.+|++
T Consensus         17  NvTL~g~~~~~v~Wyr~~~~~~~~~LC~~~~~~------~~C~~~nLtL   59 (88)
T E3TMR2_ADE16/1    17  TCTLQGPQEGHVTWWRIYDNGGFARPCDQPGTK------FSCNGRDLTI   59 (88)
T ss_pred               cEEEeCCCCCCeEEEEeccCCCcccccccCCCe------eEeCCCCeEE
```

>6JXR_n T-cell surface glycoprotein CD3 zeta; IMMUNE SYSTEM Homo sapiens
Probability: 62.4%,   E-value: 84,   Score: 22.12,   Aligned cols: 126,   Identities: 11%,   Similarity: 0.052,

```
Q Q_9601142         19  VQYNTTFQYTIDGEYK---KVEWKYNNTHLICSDGEVYEQFN------TTVKCDNISLVFDIANVTLPNVTIECKTKKGE   89 (148)
Q Consensus         19  ~~~n~t~~~tv~g~yk---~VeWk~N~s~~iCSdG~vyq~fn------~t~~Cdn~tL~~~~~~vs~p~vtieck~~~G~   89 (148)
                        ...+.+++-.+.+.+.   .+.|.++.....-+.........      ..-..-..+|++.....+-..-.+.|.+.+..
T Consensus        135  ~g~~~~l~C~~~~~~~~~~~~~~W~~~~~~~~~~~~~~~~~~~~~~~~~~~~~~~~l~i~~~~~~d~~g~y~C~~~~~~   214 (291)
T 6JXR_n           135  HTQKATLVCLATGFYPDHVELSWWVNGKEVHSGVSTDPQPLKEQPALNDSRYCLSSRLRVSATFWQNPRNHFRCQVQFYG   214 (291)

Q Q_9601142         90  ASSVTFNNTITVVTTHSPTTNPSKSLIPSTKRH----------YYFLLLAFIPPAWFAVLIIYYV   144 (148)
Q Consensus         90  ~~~~~vnn~~~~~tt~~p~~~p~~sLiPSTKR~----------yY~L~lafi~paw~~V~iihYv   144 (148)
                        ................+.......--+.....          .+.+.++++.-..++++++-.+
T Consensus        215  ~~~~~~~~~~~~~~~~~~~~~~~~~~~~~~~~~~~~~~~~~~~~~~~~~~~~~~~~~~~~~~i~~~~   279 (291)
T 6JXR_n  215          LSENDEWTQDRAKPVTQIVSAEAWGRADCGFTSESYQQGVLSATILYEILLGKATLYAVLVSALV   279 (291)
```

>3OQ3_B Interferon alpha-5, IFN-alpha/beta binding protein; Ectromelia, Mousepox Virus, Moscow strain; Mus musculus
Probability: 51.52%,   E-value: 150,   Score: 21.59,   Aligned cols: 82,   Identities: 12%,   Similarity: 0.143,

```
Q Q_9601142         19  VQYNTTFQYTIDGEYK-----KVEWKYNNTHLICSDGEVYEQFNTTVKCDNISLVFDIANVTLPNVTIECKTKKGEASSV   93 (148)
Q Consensus         19  ~~~n~t~~~tv~g~yk-----~VeWk~N~s~~iCSdG~vyq~fn~t~~Cdn~tL~~~~~~vs~p~vtieck~~~G~~~~~   93 (148)
                        .....+++-.+.|...     .+.|.+++...+-.+...+........-.+.+|++.-....--.-...|.+.|+.....
T Consensus        242  ~g~~~~l~C~~~~~~~~~~~~~~~~~~~W~~~~~~~~~~~~~~~~~~~~~~~~~~~~~~~~~~~l~i~~~~~~d~G~~Y~C~a~n~~g~~~   321 (329)
T 3OQ3_B           242  IGEPANITCTAVSTSLLVDDVLIDWENPSGWIIGLDFGVYSILTSSGGITEATLYFENVTEEYIGNTYTCRGHNYYFDKT   321 (329)

Q Q_9601142         94  TFNNTIT   100 (148)
Q Consensus         94  ~vnn~~~   100 (148)
                        .-.++..
T Consensus        322  ~~~~l~v   328 (329)
T 3OQ3_B           322  LTTTVVL   328 (329)
```
