# Supplementary material for: Novel Immunoglobulin Domain Proteins Provide Insights into Evolution and Pathogenesis of SARS-CoV-2-Related Viruses
Source: mBio. 2020 May 29;11(3):e00760-20. doi: 10.1128/mBio.00760-20 (PMC7267882; doi:10.1128/mBio.00760-20)
Supplement: TABLE S2 [file mBio.00760-20-st002.docx]

**Table S2 Summary of representatives of viral Ig domain proteins which were identified in this study**

| Family | Genus | Organism | NCBI ID | pfam ID | Domain Famliy | Presence of Signal Peptide (Y/N)* | Number of Ig-like domain | Number of TM region** | Distinct Relative PDB Structure^#^ |
| --- | --- | --- | --- | --- | --- | --- | --- | --- | --- |
| Coronaviridae | Beta-coronavirus | SARS-CoV-2 | YP_009724395.1 | PF08779 | SARS_X4 | Y | 1 | 1 | 1XAK_A |
|  |  |  | YP_009724396.1 | PF12093 | Corona_NS8 | Y | 1 | 0 | 1XAK_A |
|  |  | SARS-CoV | NP_828857.1 | PF08779 | SARS_X4 | Y | 1 | 1 | 1XAK_A |
|  |  |  | NP_828876.1  NP_828877.1 | PF08779 | Corona_NS8 | Y | 1 | 0 | 1XAK_A |
|  | Alpha-coronavirus | Bat coronavirus | QBP43259.1 | n/a | Adeno_E3_CR1-like | Y | 1 | 1 | 5XMZ_A |
|  |  |  | QBP43265.1 | PF08779 | SARS_X4 | Y | 1 | 1 | 1XAK_A |
| Adenoviridae | Mast-adenovirus | Human adenovirus 7d | AAF14132.1 | PF02440 | Adeno_E3_CR1 | Y | 1 | 1 | 6JXR_d |
|  |  | Human adenovirus 23 | AFK92306.1 | PF02440 | Adeno_E3_CR1 | Y | 3 | 1 | 3J8F_7 |
|  |  | Human adenovirus 21 | AAW33363.1 | PF04881 | Adeno_GP19K | Y | 1 | 1 | 5IRO_P |
| Herpesviridae | Mardivirus | Gallid alphaherpesvirus 2 | YP_001034013.1 | PF02480 | Herpes_gE | Y | 1 | 1 | 2GJ7_F |
|  |  |  | YP_001034012.1 | PF01688 | Herpes_gI | Y | 1 | 1 | 5OR7_C |
|  |  |  | YP_001033973.1 | PF02124 | Marek_A | Y | 3 | 1 | 3J8F_7 |
|  | Simplexvirus | Macacine alpha-herpesvirus 1 | NP_851925.1 | PF01537 | Herpes_glycop_D | Y | 1 | 1 | 4MYV_A |
|  | Rhadinovirus | Human gamma-herpesvirus 8 | YP_001129350.1 | PF02960 | K1 | Y | 2 | 1 | 5D6D_C |
|  | Cytomegalo-virus | Panine beta-herpesvirus 2 | NP_612760.1 | PF16758 | UL141 | Y | 1 | 1 | 4JM0_B |
|  |  |  | NP_612778.1 | PF05963 | Cytomega_US3 | Y | 1 | 1 | 1IM3_P |
|  |  | Human beta-herpesvirus 5 | ABV71546.1 | PF17622 | UL16 | Y | 1 | 1 | 2WY3_B |
|  |  | Aotine beta-herpesvirus 1 | YP_004940175.1 | PF08001 | CMV_US | Y | 1 | 2 | 1IM3_P |
| Poxviridae | Orthopoxvirus | Variola virus | NP_042191.1 | PF08204 | V-set_CD47 | Y | 1 | 5 | 5OR7_C |
|  |  | Ectromelia virus | 3OQ3_B | PF13895 | ig | Y | 3 | 0 | 3OQ3_B |
| Phenuiviridae | Goukovirus | Cumuto virus | YP_009664616.1 | PF07245 | Phlebovirus_G2 | Y | 4 | 1 | 6F8P_A  6EGU_B |

* Signal Peptide Prediction was conducted by SignalP-5.0 program (1).

** Transmembrane (TM) region predictions were conducted by TMHMM Server (2).

# The PDB structures which display similarity with the respective viral Ig domains identified by profile-profile comparisons (3).

**REFERENCES**

1. Armenteros JJA, Tsirigos KD, Sønderby CK, Petersen TN, Winther O, Brunak S, von Heijne G, Nielsen H. 2019. SignalP 5.0 improves signal peptide predictions using deep neural networks. Nature biotechnology 37:420-423.

2. Krogh A, Larsson B, Von Heijne G, Sonnhammer EL. 2001. Predicting transmembrane protein topology with a hidden Markov model: application to complete genomes. Journal of molecular biology 305:567-580.

3. Söding J, Biegert A, Lupas AN. 2005. The HHpred interactive server for protein homology detection and structure prediction. Nucleic acids research 33:W244-W248.
