# Supplementary material for: Novel Immunoglobulin Domain Proteins Provide Insights into Evolution and Pathogenesis of SARS-CoV-2-Related Viruses
Source: mBio. 2020 May 29;11(3):e00760-20. doi: 10.1128/mBio.00760-20 (PMC7267882; doi:10.1128/mBio.00760-20)
Supplement: FIG S2 [file mBio.00760-20-sf002.pdf]

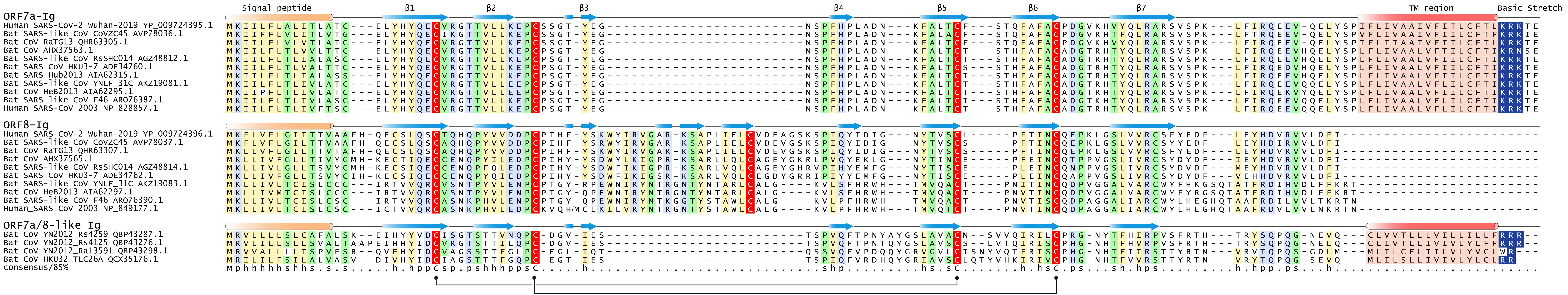

ORF7a-Ig
Signal peptide
β1
β2
β3
β4
β5
β6
β7
TM region
Basic Stretch
Human SARS-CoV-2 Wuhan-2019 YP_009724395.1
Bat SARS-like CoV CovZC45 AVP78036.1
Bat CoV RaTG13 QHR63305.1
Bat CoV AHX37563.1
Bat SARS-like CoV RsSHC014 AGZ48812.1
Bat SARS CoV HKU3-7 ADE34760.1
Bat SARS Hub2013 AIA62315.1
Bat SARS-like CoV YNLF_31C AKZ19081.1
Bat CoV HeB2013 AIA62295.1
Bat SARS-like CoV F46 ARO76387.1
Human SARS-CoV 2003 NP_828857.1
ORF8-Ig
Human SARS-CoV-2 Wuhan-2019 YP_009724396.1
Bat SARS-like CoV CovZC45 AVP78037.1
Bat CoV RaTG13 QHR63307.1
Bat CoV AHX37565.1
Bat SARS-like CoV RsSHC014 AGZ48814.1
Bat SARS CoV HKU3-7 ADE34762.1
Bat SARS-like CoV YNLF_31C AKZ19083.1
Bat CoV HeB2013 AIA62297.1
Bat SARS-like CoV F46 ARO76390.1
Human_SARS CoV 2003 NP_849177.1
ORF7a/8-like Ig
Bat CoV YN2012_Rs4259 QBP43287.1
Bat CoV YN2012_Rs4125 QBP43276.1
Bat CoV YN2012_Ra13591 QBP43298.1
Bat CoV HKU32_TLC26A QCX35176.1
consensus/85%
